# A comprehensive computational analysis to explore the importance of SIGLECs in HCC biology

**DOI:** 10.1186/s12876-023-02672-z

**Published:** 2023-02-18

**Authors:** Senbang Yao, Wenjun Chen, Tingting Chen, He Zuo, Ziran Bi, Xiuqing Zhang, Lulian Pang, Yanyan Jing, Xiangxiang Yin, Huaidong Cheng

**Affiliations:** 1 Department of Oncology, The Second Affiliated Hospital of Anhui Medical University, Hefei, 230601 Anhui China; 2 Department of Oncology, Anhui Medical University, Hefei, Anhui China; 3 Department of Oncology, Anhui Chest Hospital, Hefei, Anhui China; 4 The Third School of Clinical Medicine, Southern Medical University, Shenzhen, Guangdong China; 5 Department of Oncology, Shenzhen Hospital of Southern Medical University, Shenzhen, 518000 Guangdong China

**Keywords:** Hepatocellular carcinoma, Sorafenib, Prognostic markers, Immunotherapy, Tumour progression

## Abstract

**Background:**

Hepatocellular carcinoma (HCC) is an aggressive, malignant cancer with a complex pathogenesis. However, effective therapeutic targets and prognostic biomarkers are limited. Sorafenib provides delaying cancer progression and survival improvement in advanced HCC. But despite 10 years of research on the clinical application of sorafenib, predictive markers for its therapeutic effect are lacking.

**Methods:**

The clinical significance and molecular functions of SIGLEC family members were assessed by a comprehensive bioinformatic analysis. The datasets included in this study (ICGC-LIRI-JP, GSE22058 and GSE14520) are mainly based on patients with HBV infections or HBV-related liver cirrhosis. The TCGA, GEO, and HCCDB databases were used to explore the expression of SIGLEC family genes in HCC. The Kaplan–Meier Plotter database was used to evaluate relationships between the expression levels of SIGLEC family genes and prognosis. Associations between differentially expressed genes in the SIGLEC family and tumour-associated immune cells were evaluated using TIMER.

**Results:**

The mRNA levels of most SIGLEC family genes were significantly lower in HCC than in normal tissues. Low protein and mRNA expression levels of SIGLECs were strongly correlated with tumour grade and clinical cancer stage in patients with HCC. Tumour-related SIGLEC family genes were associated with tumour immune infiltrating cells. High SIGLEC expression was significantly related to a better prognosis in patients with advanced HCC treated with sorafenib.

**Conclusions:**

SIGLEC family genes have potential prognostic value in HCC and may contribute to the regulation of cancer progression and immune cell infiltration. More importantly, our results revealed that SIGLEC family gene expression may be used as a prognostic marker for HCC patients treated with sorafenib.

**Supplementary Information:**

The online version contains supplementary material available at 10.1186/s12876-023-02672-z.

## Background

Malignant tumours pose a serious threat to public health worldwide, including hepatocellular carcinoma (HCC), which is caused by various pathogenic factors, such as alcohol use and hepatitis [1]. HCC is currently the third-leading cause of cancer mortality [2]. Although some patients with HCC are cured by resection or liver transplant, overall survival (OS) is still low. The poor prognosis in HCC can be explained by the frequent diagnosis at a late stage [3]. The majority of HCC patients develop on the background of cirrhosis[4] and then generate a regional milieu that predisposes them to HCC[5]. Sorafenib, an inhibitor of multitarget tyrosine kinases, is used for the treatment of advanced-stage HCC [6]. However, the median life expectancy of patients with HCC treated with sorafenib is only 1 year. Despite 10 years of research on the clinical application of sorafenib, there are still no confirmed predictive factors for its therapeutic effect [7–9].

Sialic-acid-binding immunoglobulin-like lectins (SIGLECs) are a family of sialic acid-recognition proteins expressed primarily on leukocytes involved in the fine-tuning of leukocyte activities [10]. The roles of SIGLECs in neoplasms have already been described, and therapeutic agents directed at SIGLECs are under investigation [11]. SIGLEC are mainly expressed on immune cell surfaces. SIGLECs have been summarized in a review that they could play diverse regulatory functions in the tumor microenvironment (TME), while participating in HCC progression by various mechanisms, such as regulating cancer metastasis, and promoting cancer immune escape [12]. Thus, SIGLECs can become a target for regulating immunological processes [13]. The connection between SIGLEC expression and prognosis has been evaluated extensively. Previous studies have shown that SIGLECs family genes play important functions in HCC. SIGLEC2 is the best characterized member of the SIGLEC family [14], and its down-regulation has been found to predict worse overall survival of HBV-related HCC [15]. Wu et al. found that CD24-SIGLEC10 interaction may be involved in immune response, which could potentially become a therapeutic target for HCC patients [16]. Wang et al. reported that reduced SIGLEC7 expression could cause NK cell dysfunction in HCC patients [17]. Yamada reported an association between SIGLEC7 expression and prognosis in colorectal cancer (CRC) [18]. Wang et al. identified SIGLEC15 as a prospective target for tumour immunotherapy [19]. Ye et al. have shown that SIGLEC family genes may manipulate the tumour microenvironment via the chemokine axis and are associated with prognosis [20]. However, the function of SIGLEC family members in HCC remains uncertain, and comprehensive explorations of its biological effects and molecular pathways are essential for developing new prognostic indicators and therapeutic targets.

In this study, we explored this question by evaluating the expression of SIGLEC family members at the mRNA and protein levels using The Cancer Genome Atlas (TCGA), Gene Expression Omnibus (GEO), and Human Protein Atlas (HPA) databases. A multidimensional analysis was used to structure functional networks and genomic alterations associated with HCC, and its effect on tumour immunity was explored. Additionally, clinical characteristics associated with SIGLEC family genes and the prognostic value of these genes in HCC were analysed. Hence, this is a comprehensive computational analysis to explore the importance of SIGLECs in HCC biology and prognosis.

## Methods

### Differences in SIGLEC expression at the transcriptional level

Most HCC patients included in this study have a history of HBV infection or HBV-related liver cirrhosis. TCGA is a famous cancer gene project containing genomic information for 33 primary cancers and matching noncancer tissues [21]. GEPIA is a database that includes tumour and normal samples from TCGA and the Genotype-Tissue Expression (GTEx) dataset. GEPIA was used to explore the difference in SIGLEC expression between cancer and corresponding normal tissues in TCGA datasets [22]. UALCAN is a website for analysing tumour genomic data [23]. We used UALCAN to explore the correlations between the expression of SIGLEC family members and tumour staging and grading in HCC.

GEO is a public genome data storage centre for MIAME-compliant data. Biological and medical researchers can query and download gene expression profiles of interest [24]. GSE14520 [25] and GSE22058 [26] datasets were obtained from the GEO database. In addition, the International Cancer Genome Consortium (ICGC) was used to explore differences in SIGLEC gene expression at the transcriptional level. The specific dataset was ICGC-LIRI-JP, which contains 231 liver cancer samples, RNA-seq data, and clinical information [27].

### Differential SIGLEC expression at the protein level

To investigate SIGLEC expression in HCC at the protein level, HPA was used to explore the immunohistochemical data for SIGLEC protein expression in HCC and normal tissues [28]. The SIGLEC protein expression levels in normal hepatic tissues were explored in the tissue module, which contains abundant tissue protein expression information for healthy humans.

### Molecular network structure and functional clustering of SIGLECs in HCC

GeneMANIA is a platform for gene function speculation, analyses of gene lists, and gene prioritization [29]. In this study, SIGLEC family members were submitted to GeneMANIA to explore correlations with other genes, including coexpressed genes, associated protein domains, interactions, colocalizing genes, and genetic interactions.

WebGestalt [30] (http://www.webgestalt.org) is a tool for functional enrichment analyses. Kyoto Encyclopedia of Genes and Genomes (KEGG) pathway [31–33] and Gene Ontology (GO) functional analyses of SIGELCs and correlated genes were performed using WebGestalt. GO functional enrichment was evaluated in the biological process (BP), cellular component (CC), and molecular function (MF) categories. The enriched pathways were evaluated by KEGG pathway analysis. Metascape is a platform used for inclusive gene list annotation and source analysis for investigational biologists. We further analysed gene enrichment of the SIGLEC family using DisGeNET [34] in Metascape [35] (https://metascape.org/gp/index.html).

### Analysis of tumour immune invasion

Tumour immune invasion was explored by using Tumour Immune Estimation Resource (TIMER) [36], which can be used for integrated analyses of cancer-infiltrating immune cells. SIGLECs-related immune infiltration was explored in HCC using TIMER. SIGLEC mRNA expression in HCC and relationships between SIGLEC expression and immune cell abundances were explored, including CD4+ T cells, B cells, and CD8+ T cells. The purity-corrected partial Spearman’s rho values were obtained. Using TIMER, the relationships between SIGLEC family member expression levels and PD1, PD-L1, and CTLA4 levels were evaluated.

### Survival and mutation analysis of SIGLEC in HCC

cBioPortal is a cancer database for data integration, data mining, and visualization [37]. Gene alterations in SIGLECs were visualized using OncoPrint. cBioPortal was used to analyse SIGLEC alterations in TCGA-HCC cases. The analytical parameters included mRNA expression, copy number variants, mutations, and survival.

In this study, the prognostic value of SIGLEC family gene expression in HCC was explored using Kaplan–Meier plotter. This database is used to analyse correlations of the expression levels of various genes and survival in multiple kinds of cancer. The data sources include GEO, TCGA, and EGA. A Kaplan–Meier survival analysis was performed to contrast two patient cohorts. The HR with 95% confidence interval and log-rank *P value* were assessed. Clinical information in the database is regularly updated and maintained [38].

### Steps of data extraction and analysis in different databases

HCCDB [39] (http://lifeome.net/database/hccdb/home.html). Time of login: April 2022. Version: HCCDB 2018. Data source of HCCDB: GEO data series, ICGC and TCGA-LIHC. The box plot of HCCDB18 (ICGC-LIRI-JP) included the SIGLECs differential expression was shown in Fig. 3A. And Fig. 3B, C shows the results of HCCDB 1 (GSE22058) and HCCDB 16 (GSE14520). A heatmap of SIGLEC family expression in tissue and prognosis was generated (Fig. 3D). The data of the datasets contained in the HCCDB database could be downloaded, including the number of adjacent samples and the number of HCC samples. This is where the content for Table 1 comes from.

**Table 1:** Information on HCCDB datasets

P value	Number of adjacent samples	Number of HCC samples	Source
SIGLEC1			
2.340e−30	97	100	GSE22058
1.53e−6	52	115	GSE76427
SIGLEC2			
2.824e−2	82	80	GSE10143
SIGLEC3			
4.231e−3	177	212	ICGC-LIRI-JP
SIGLEC4			
2.82e−6	49	356	TCGA-LIHC
SIGLEC5			
2.155e−4	168	228	GSE63898
SIGLEC6			
1.890e−15	220	225	GSE14520
SIGLEC7			
2.430e−34	97	100	GSE22058
SIGLEC8			
1.7e−6	49	356	TCGA-LIHC
SIGLEC9			
8.423e−4	52	115	GSE76427
SIGLEC10			
1.251e−2	80	81	GSE54236
SIGLEC11			
5.760e−37	193	240	GSE36376
SIGLEC14			
3.540e−22	168	228	GSE63898
SIGLEC15			
7.336e−2	220	225	GSE14520
SIGLEC16			
3.85e−6	52	115	GSE76427

The Human Protein Atlas [28] (https://www.proteinatlas.org). Time of login: August 2021. Version: HPA 21.1. Data source of The Human Protein Atlas: A Swedish-based program. The immunohistochemical images of liver cancer tissue and normal liver tissue were obtained and the protein expression of SIGELC family members in HCC (Fig. 5) can be generated in HPA.

GeneMANIA [29] (http://genemania.org). Time of login: August 2021. Version: GeneMANIA 2.6. Data source of GeneMANIA: Genomics and proteomics data based on instantly updated publicly available databases. The network of SIGLECs can be generated in GeneMANIA (Fig. 6A).

PINA [40, 41] (https://omics.bjcancer.org/pina/home.action). Time of login: August 2021. Version: PINA v3.0. Data source of PINA: TCGA-LIHC. The network of SIGLECs in HCC can be generated in PINA (Fig. 6B).

WebGestalt [30] (http://www.webgestalt.org). Time of login: August 2021. Version: WebGestalt 2019. Data source of WebGestalt: WebGestalt supports 342 gene identifiers and 155 175 functional categories, as well as user-uploaded functional databases. The data were derived from large-scale omics studies. The functional enrichment analysis of SIGLEC genes (Fig. 6C–F). The multiple test correction was perfomed in Gene set enrichment analysis by WebGestalt.

Metascape [35] (https://metascape.org/gp/index.html). Time of login: August 2021. Version: Metascape 2019. Data source of Metascape: A broad set of instantly updated biological databases. Summary of enrichment analysis in DisGeNET (Fig. 6G).

TIMER [36] (https://cistrome.shinyapps.io/timer). Time of login: August 2021. Version: TIMER 1.0. Data source of TIMER: TCGA-LIHC. The correlation results between PD-1, PD-L1, CTLA-4 and SIGLEC genes (Fig. 7). The correlation results between T-cell infiltration, B-cell infiltration and SIGLEC genes (Table 4).

TISIDB [42] (http://cis.hku.hk/TISIDB/index.php). Time of login: August 2021. Version: TISIDB 2019. Data source of TISIDB: TCGA-LIHC. The correlations between SIGLEC mRNA expression levels and tumour grade in HCC (Fig. 8).

GEPIA [22] (http://gepia.cancer-pku.cn). Time of login: August 2021. Version: GEPIA 1.0. Data source of GEPIA: TCGA-LIHC. The correlations between SIGLEC mRNA expression levels and cancer stage in HCC (Figs. 4, 9).

cBioPortal [43] (https://www.cbioportal.org). Time of login: August 2021. Version: cBioPortal v5.1.0. Data source of cBioPortal: Multidimensional cancer genomics datasets. The overview of 279 SIGLEC gene mutations (Fig. 10A). The SIGLEC gene alteration frequencies (Fig. 10B). The survival in SIGLEC mutation group (Fig. 10C, D).

Kaplan–Meier Plotter [38] (http://kmplot.com). Time of login: August 2021. Version: Kaplan–Meier Plotter 2021. Data source of Kaplan–Meier Plotter: TCGA-LIHC, GSE20017 and GSE9843. The survival results correlated with SIGLEC expression (Fig. 11). The effect of SIGLEC expression on survival and prognosis in patients with advanced HCC treated with sorafenib (Fig. 12).

### Statistical analysis

**Fig. 1:**
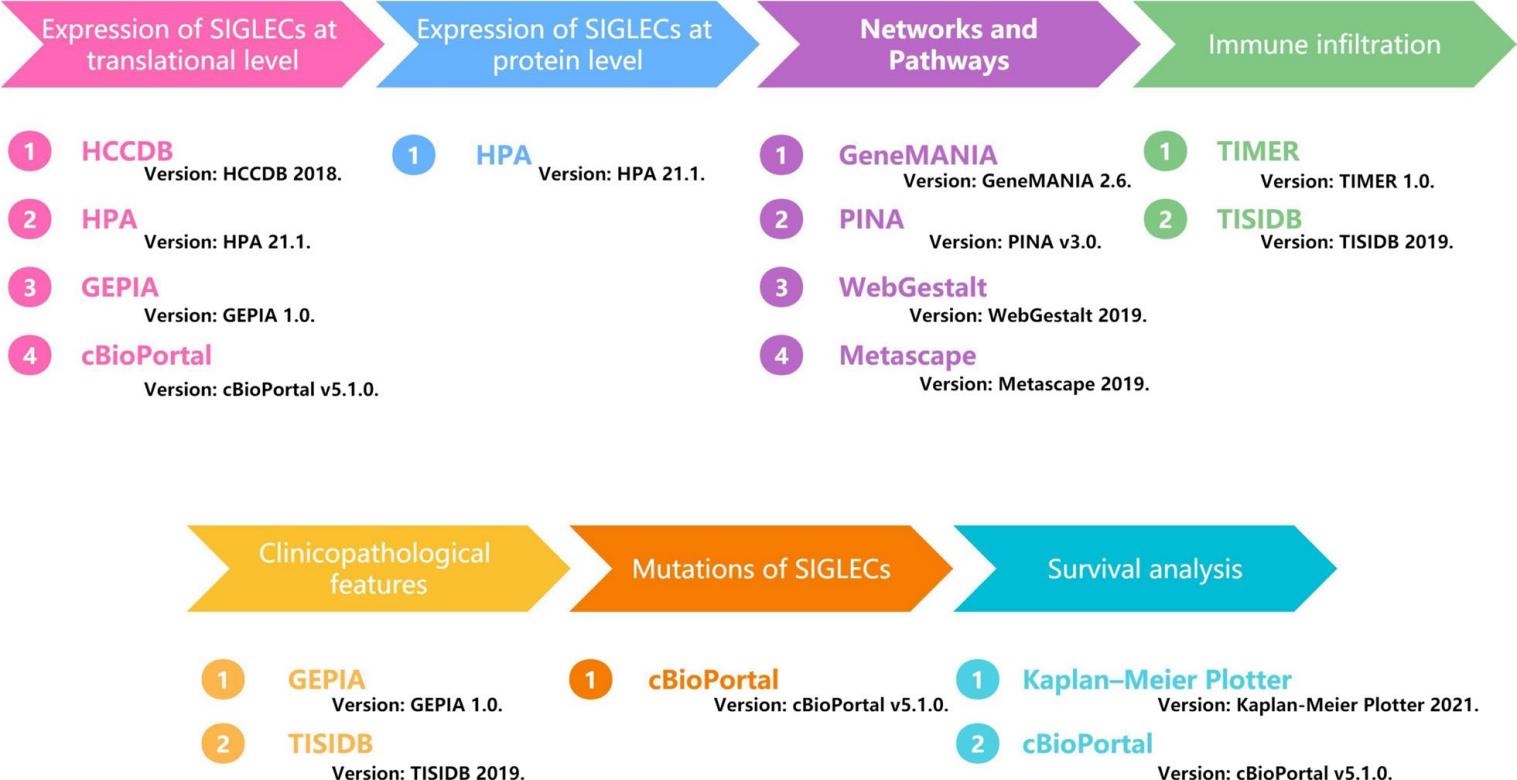
Flow chart of analytical pipeline

SIGLECs expression levels were compared by *t*-tests. Kaplan–Meier analyses were used to evaluate differences in prognosis. OS was defined as the time interval from diagnosis to death. Recurrence-free survival (RFS) was defined as the time interval from diagnosis to recurrence or last follow‐up. Progression-free survival (PFS) was defined as the time from random assignment in a clinical trial to disease progression or death from any cause. Log-rank *P* < 0.05 revealed a significant difference in prognosis. One-way ANOVA, Wilcoxon signed-rank tests, and logistic regression were used to analyse the correlations between clinicopathologic features and SIGLEC expression levels. The relationships between SIGLEC gene expression levels and immune scores were calculated by using Spearman methods. The data visualization were produced using the graphics package ggplot2 [44, 45] (Fig. 1).

## Results

### SIGLECs family genes are expressed at low levels in HCC

**Fig. 2:**
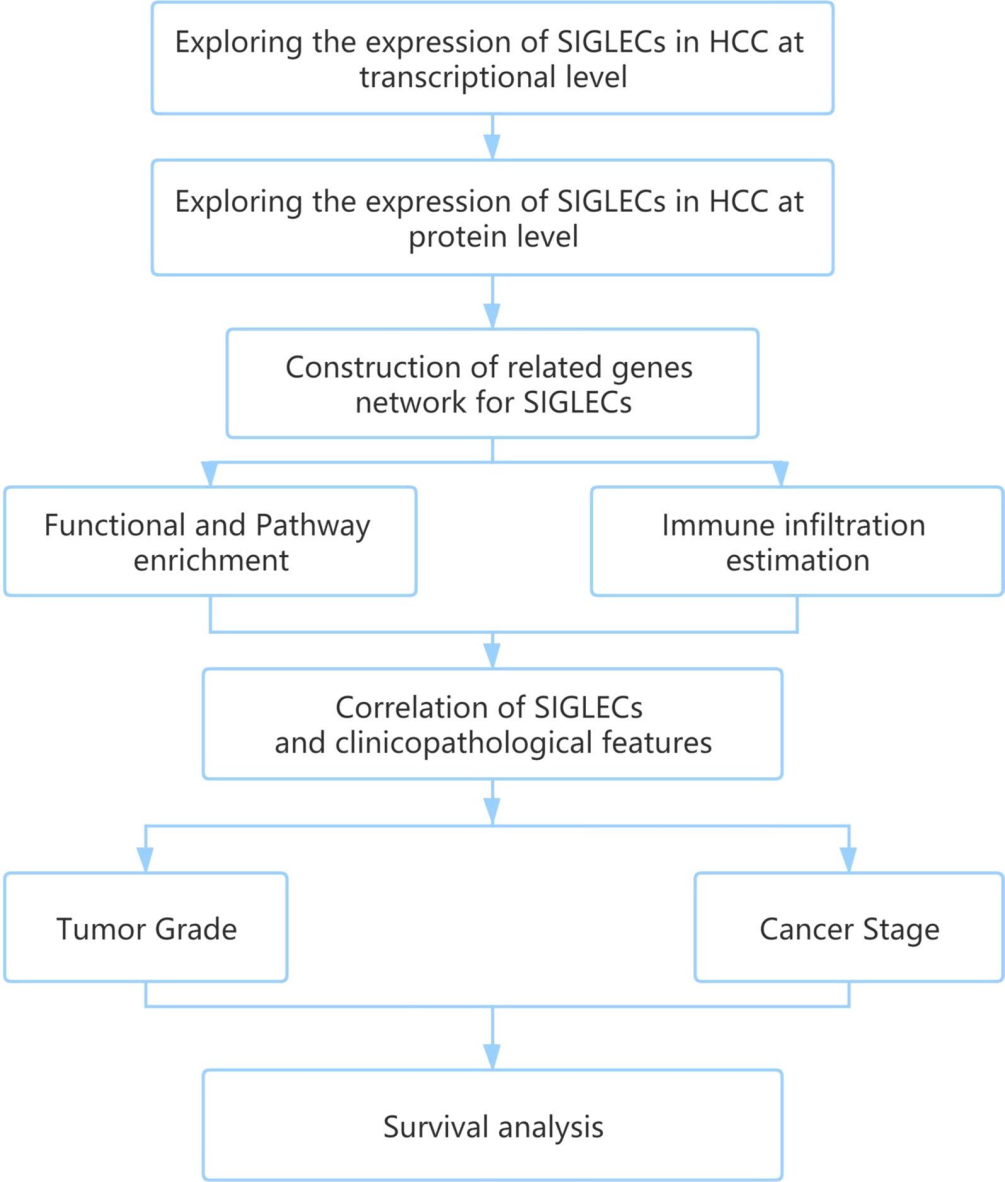
Flow chart of the analysis process in this study

An overview of the research process is shown in Fig. 2.

**Fig. 3:**
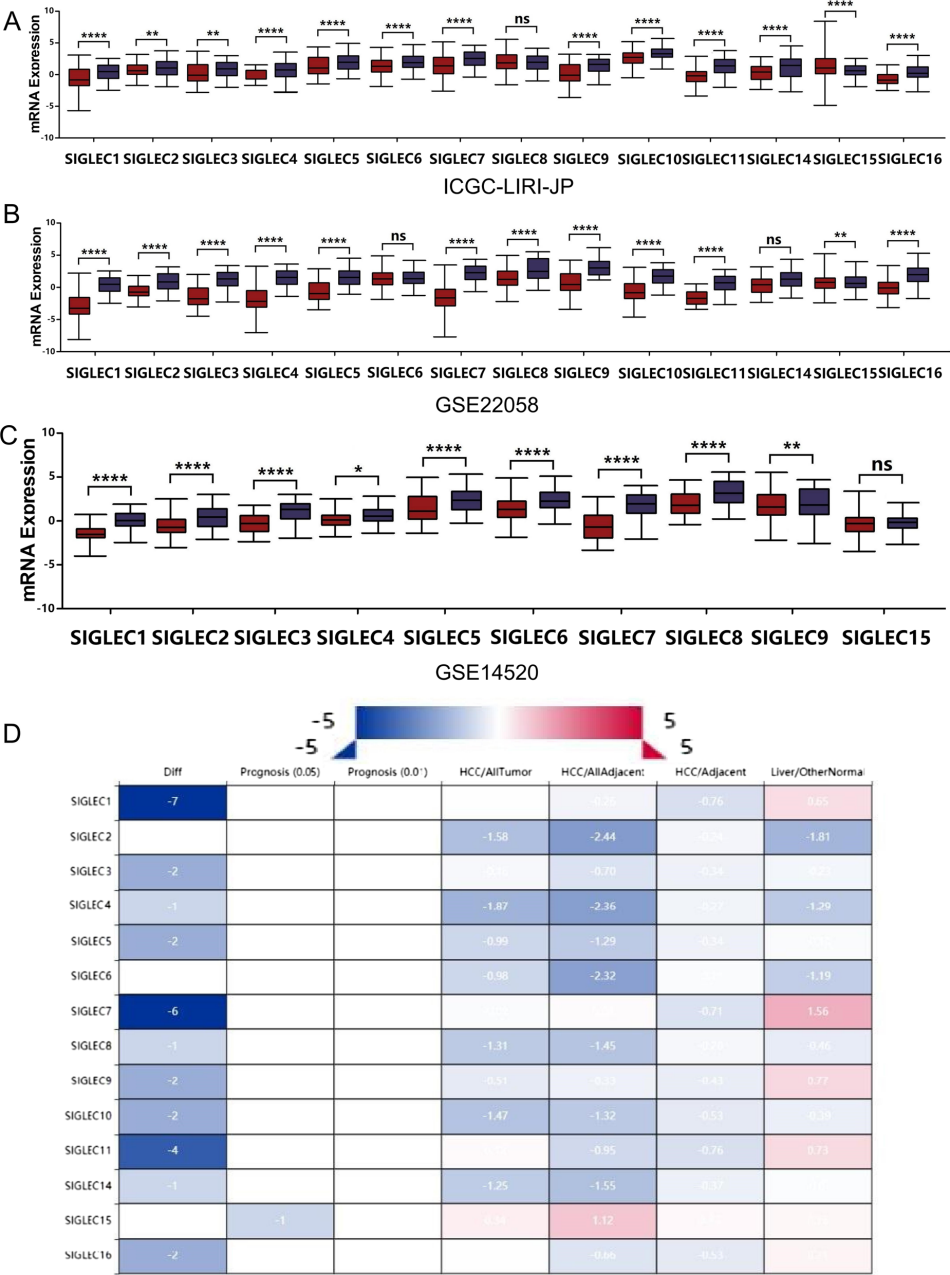
Transcriptional expression of SIGLEC family members in ICGC and GEO databases. Expression levels of SIGLEC family genes in cancer and normal tissues based on data from the **A** ICGC database, **B** GEO database (GSE22058), and **C** GEO database (GSE14520). **D** Heatmap representation of differential expression. Diff indicates the number of differentially expressed genes, blue indicates downregulation, and red indicates upregulation (HCCDB)

To explore SIGLEC family member expression in HCC, the ICGC and GEO databases were used for comparisons of expression levels between cancer tissues and correlated normal tissues. First, in the ICGC-LIRI-JP datasets, the levels of SIGLEC family members were significantly lower in HCC tissues than in adjacent nontumor tissues (Fig. 3A). Furthermore, based on the GSE22058 dataset, the levels of SIGLEC family members were lower in HCC tissues than in paired adjacent nontumor tissues (Fig. 3B). Similar results were obtained for the GSE14520 dataset (Fig. 3C). These results indicate that SIGLEC family mRNA expression levels were significantly downregulated in HCC tissues. A heatmap showed that the most highly differentially expressed genes included SIGLEC1 and SIGLEC7 (Fig. 3D). SIGLECs family members showed decreased expression in cancer tissues in different HCCDB datasets, and the differences were significant. Information about the datasets is summarized in Tables 1, 2 and 3.

**Table 2:** The clinical information of TCGA-HCC patients

Characteristic	Levels	Overall
n		374
CHILD–Pugh grade, n (%)	A	219 (90.8%)
	B	21 (8.7%)
	C	1 (0.4%)
	Unknown	133
AFP values (ng/ml), n (%)	≤ 400	215 (76.8%)
	> 400	65 (23.2%)
	Unknown	94
OS event, n (%)	Alive	244 (65.2%)
	Dead	130 (34.8%)
PFS event, n (%)	Alive	191 (51.1%)
	Dead	183 (48.9%)
Histologic grade, n (%)	G1	55 (14.9%)
	G2	178 (48.2%)
	G3	124 (33.6%)
	G4	12 (3.3%)
	Unknown	5
TNM stage, n (%)	Stage I	173 (49.4%)
	Stage II	87 (24.9%)
	Stage III	85 (24.3%)
	Stage IV	5 (1.4%)
	Unknown	24
AGE, n (%)	≤ 60	177 (47.5%)
	> 60	196 (52.5%)
	Unknown	1
Severity of HBV, n (%)	None	118 (49.8%)
	Mild	101 (42.6%)
	Severe	18 (7.6%)
	Unknown	137

AFP, alpha fetoprotein; OS, overall survival; PFS, progression-free survival; HBV, hepatitis B virus

**Table 3:** Details of the primary databases used in this study

Database	GEPIA/TIMER/HPA	Kaplan–Meier plotter
Source	TCGA-HCC	GSE20017/GSE9843
Number of samples	371	226
Gender		
Male	250	156
Female	121	60
TNM stage		
I	171	9
II	86	56
III	85	7
IV	5	8
Race		
White/Caucasian	184	174
Black or African-American	17	7
Asian	158	32
Death event	130	55

**Fig. 4:**
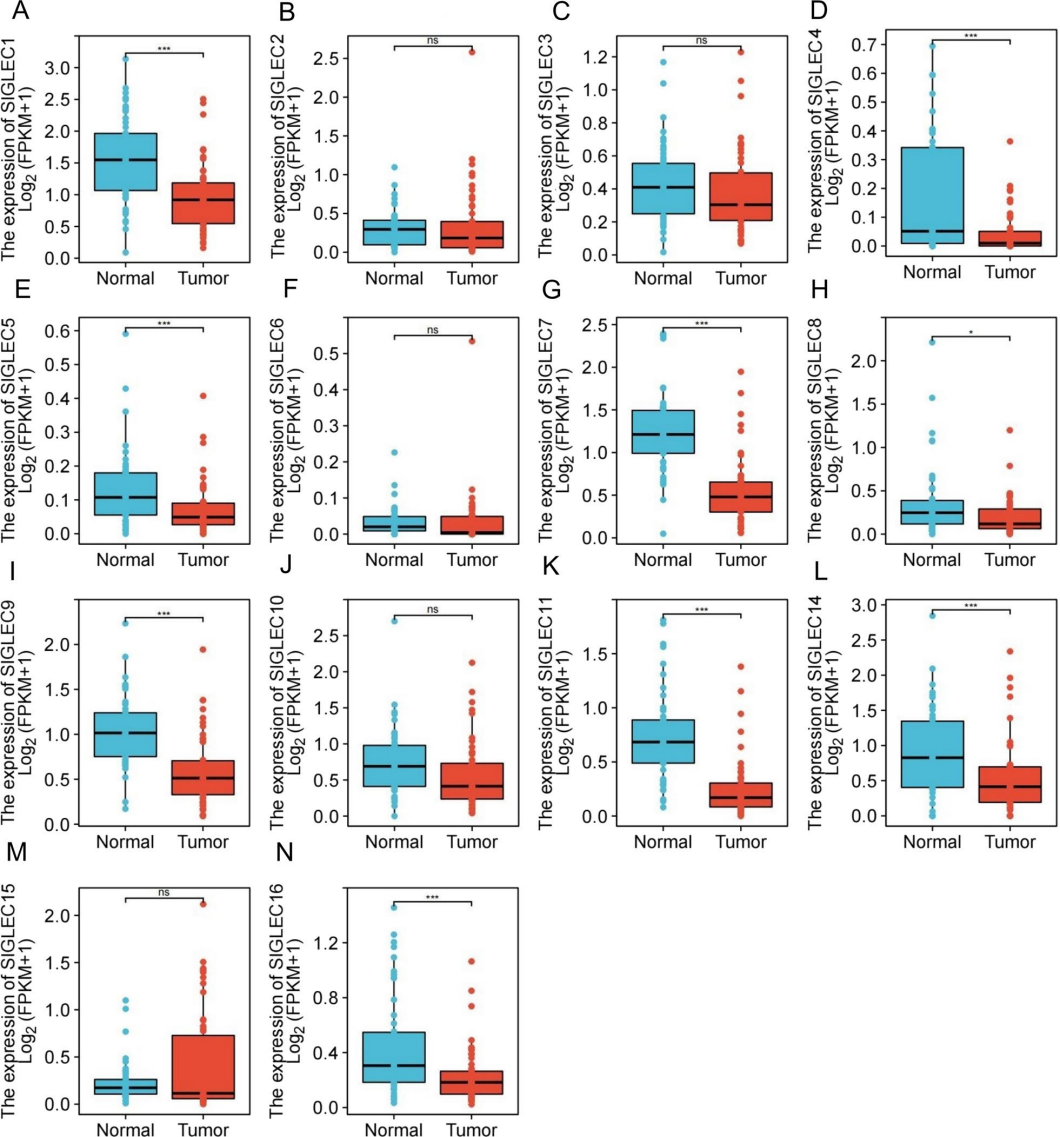
Expression levels of SIGLEC family genes in normal liver tissues and HCC tissues (TCGA database). The mRNA expression levels of most SIGLEC genes were significantly reduced in HCC (**A**–**N**). ***P < 0.001

The mRNA expression levels of SIGLEC family genes were explored using TCGA data. The expression levels of most SIGLEC genes were significantly lower in HCC tissues than in normal samples, and genes with statistically significant differences included SIGLEC1, 4, 5, 7, 8, 9, 11, 14, and 16. These results were consistent with those of the GEO analysis (Fig. 4). Since the majority of cases develop on the background of cirrhosis, SIGLECs expression in cirrhotic controls, normal healthy controls, adjacent normal controls and HCC (GSE25097) were analysed (Additional file 1: Fig. S6).

### Protein expression of SIGELC family members in HCC

**Fig. 5:**
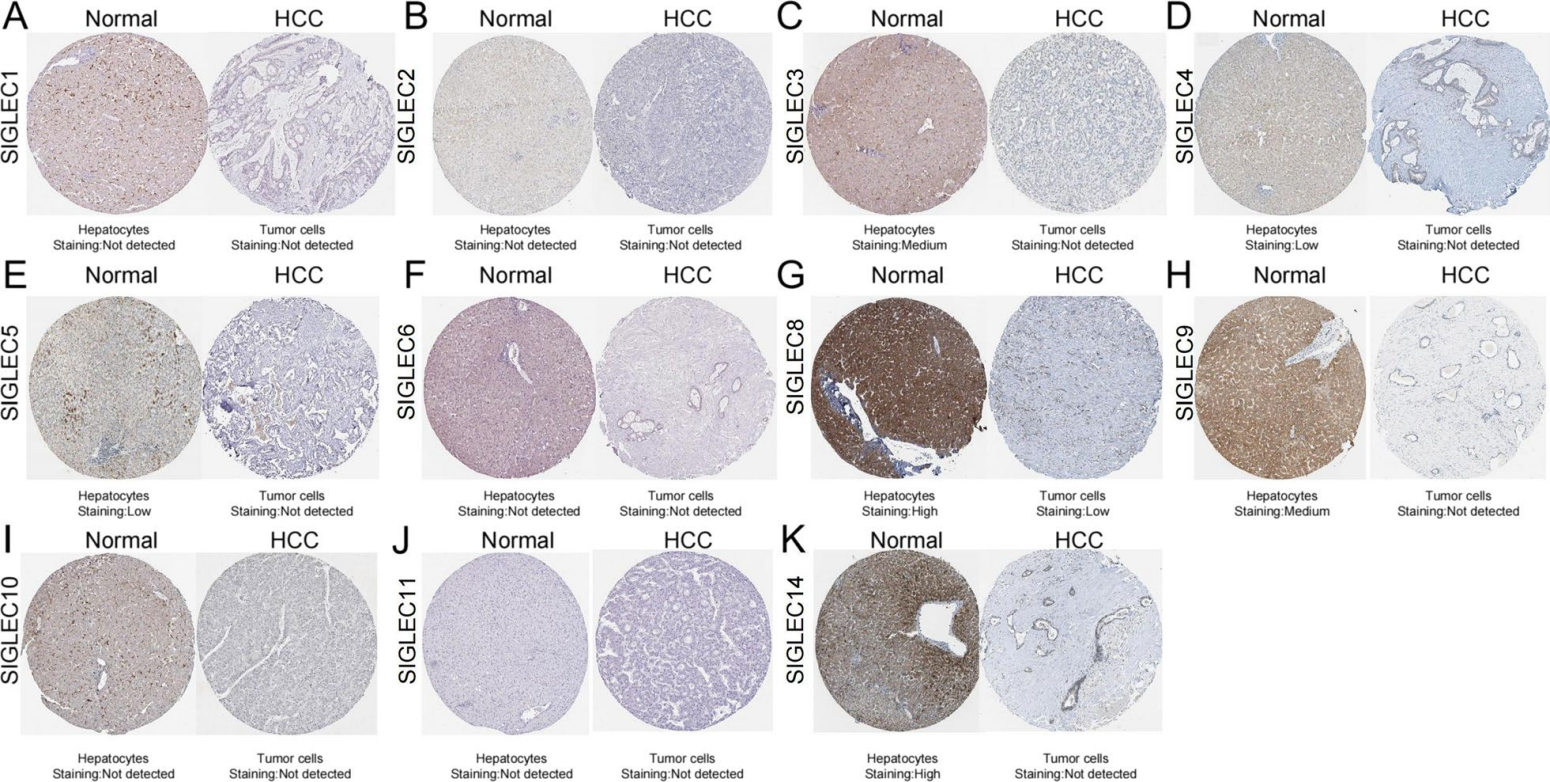
Immunohistochemical results for SIGLECs in normal liver tissues and HCC tissues (HPA database)

SIGLECs protein expression in HCC was investigated using data from the HPA. The results were similar to those of the mRNA-level analysis. SIGLECs protein expression levels were low in HCC tissues (Fig. 5). SIGLEC3, 4, 5, 8, 9, and 14 were expressed at significantly lower levels in HCC tissues than in normal tissues. SIGLEC1, 2, 6, 10, and 11 protein expression levels were low in both HCC tissues and normal liver tissues (Fig. 5). Overall, these results indicated that SIGLEC expression is significantly reduced in HCC.

### Functional enrichment analysis of SIGLEC genes in HCC

**Fig. 6:**
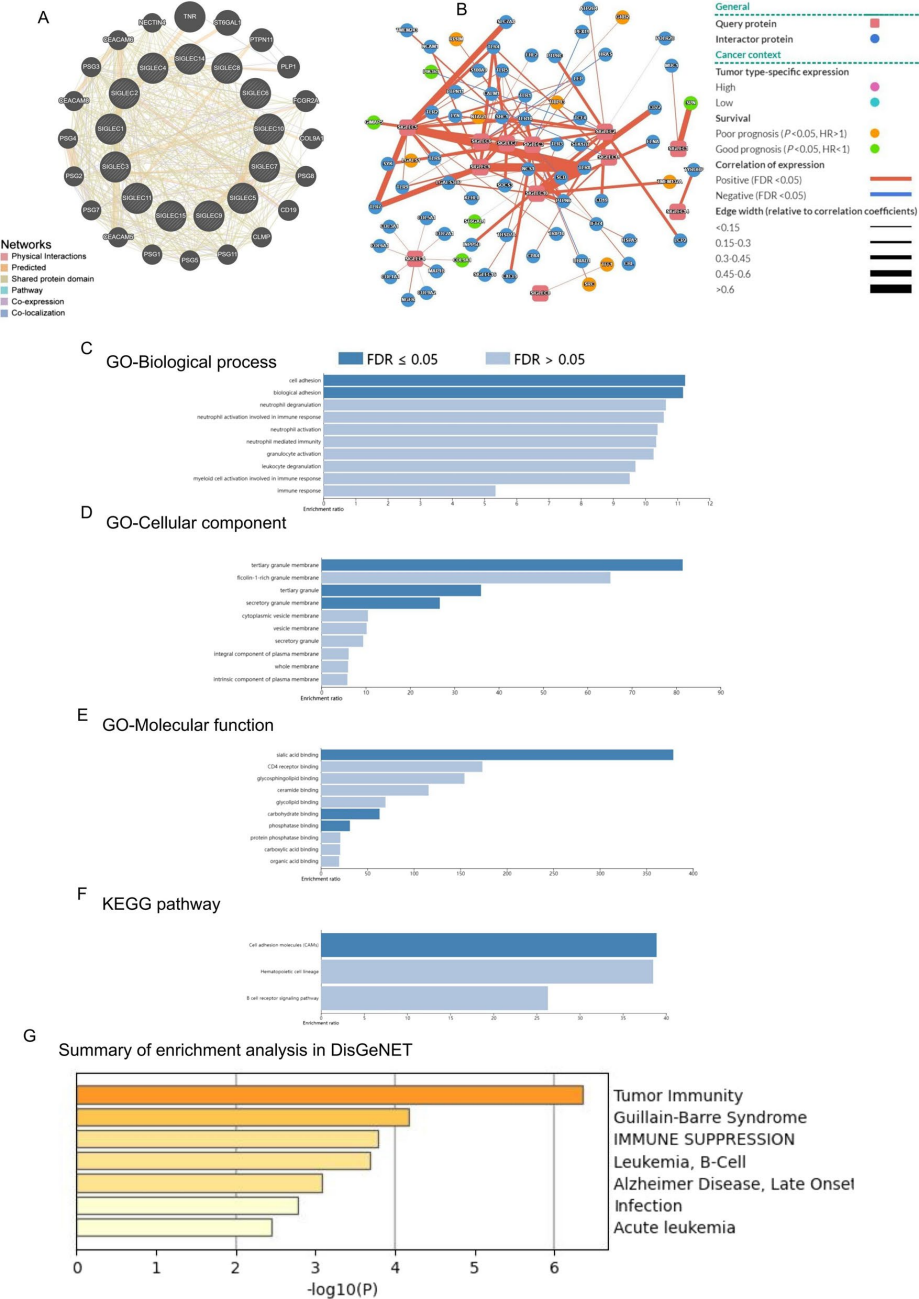
Functional enrichment of SIGLEC genes in HCC. **A** A gene network of SIGLEC and correlated genes was generated using GeneMANIA. **B** Interaction network analysis of SIGLEC family members using the PINA platform. **C** Cellular component. **D** Biological process. **E** Molecular function. **F** KEGG pathway analysis [31–33]. **G** Gene list enrichment

A network of SIGLECs and correlated genes was created using GeneMANIA (Fig. 6A) and PINA (Fig. 6B). With the functional network diagram, specific negatively and positively correlated genes that interact with SIGLEC genes were obtained.

WebGestalt was used to explore the GO and KEGG pathway enrichment. Various biological processes, such as cell adhesion, biological adhesion, and neutrophil activation, involved in the immune response were significantly related to SIGLECs in HCC (Fig. 6C). Cellular components, including tertiary granules, tertiary granule membranes, and secretory granule membranes, were identified (Fig. 6D). SIGLECs were also enriched for terms in the molecular function category (Fig. 6E), such as sialic acid binding, CD4 receptor binding, and glycosphingolipid binding.

A KEGG analysis showed that the SIGELC family was involved in various pathways [31–33], including the haematopoietic cell lineage, cell adhesion molecules (CAMs), and the B-cell receptor signalling pathway (Fig. 6F). A gene list enrichment analysis of members of the SIGLEC family was performed using Metascape. The SIGLEC family was obviously enriched in tumour immunity, Guillain–Barre syndrome, immune suppression, and other functions (Fig. 6G).

### Relationships between SIGELC family mRNA expression levels and the degree of immune cell infiltration in HCC

**Fig. 7:**
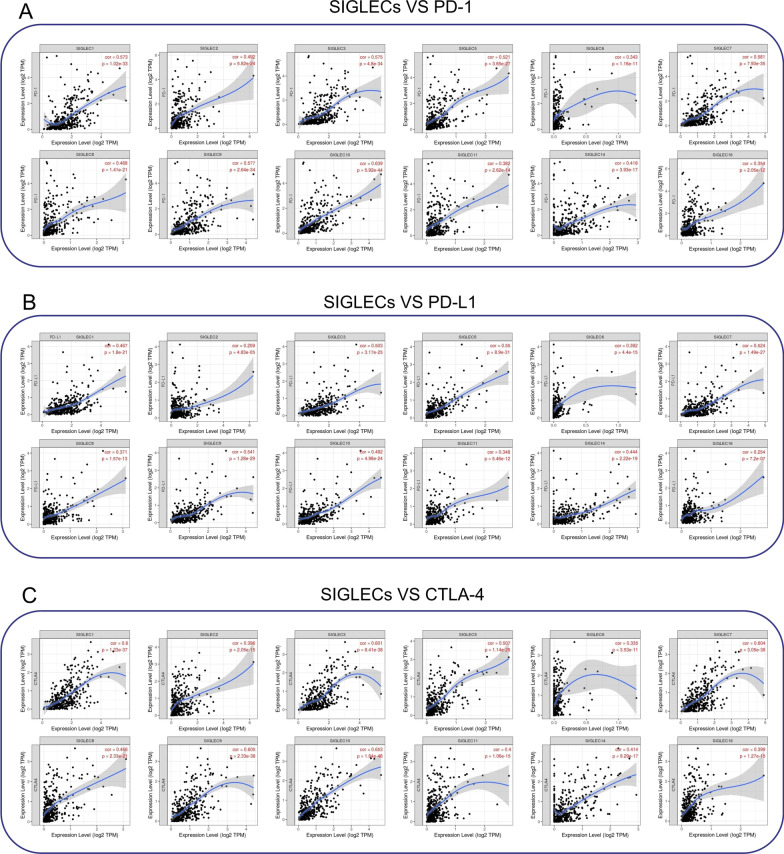
Correlations between the SIGELC family and the immune-related genes *PD-1, PD-L1*, and *CTLA4* (TCGA database). **A** Correlations between gene expression levels of SIGLECs and PD-1. **B** Correlations between the gene expression levels of SIGLECs and PD-L1. **(C)** Correlations between gene expression levels of SIGLECs and CTLA-4

We used the TIMER database to analyse the correlations between SIGLEC family members and immune infiltration in HCC. The mRNA expression levels of most SIGLECs were significantly correlated with B-cell frequencies. The mRNA expression levels of SIGLEC1, 2, 3, 5, 6, 7, 8, 9, 10, 11, 14, 15, and 16 were obviously related to CD4+ T cells. In addition, the mRNA expression levels of SIGLEC1, 2, 3, 5, 6, 7, 8, 9, 10, 11, 14, and 16 were closely related to the level of CD8+ T-cell infiltration in HCC (Table 4). Correlations between gene expression levels and levels of PD-1 (Fig. 7A), PD-L1 (Fig. 7B), and CTLA-4 (Fig. 7C) were evaluated.

**Table 4:** Association between the expression levels of SIGLEC family members and the levels of T-cell infiltration and B-cell infiltration in HCC (TCGA database)

SIGLEC	B-Cell		CD4 + T-Cell		CD8 + T-Cell	
	Cor	P value	Cor	P value	Cor	P value
SIGLEC1	0.458	**3.29E−19**	0.281	**1.22E−07**	0.535	**1.06E−26**
SIGLEC2	0.337	**1.45E−10**	0.388	**8.00E−14**	0.325	**7.85E−10**
SIGLEC3	0.561	**6.30E−30**	0.332	**2.55E−10**	0.572	**4.51E−31**
SIGLEC4	0.058	2.86E−01	0.059	2.78E−01	0.102	5.97E−01
SIGLEC5	0.550	**1.28E−28**	0.434	**3.18E−17**	0.513	**2.47E−24**
SIGLEC6	0.249	**3.01E−06**	0.279	**1.51E−07**	0.277	**1.90E−07**
SIGLEC7	0.558	**1.53E−29**	0.313	**2.78E−09**	0.597	**1.89E−34**
SIGLEC8	0.335	**1.70E−10**	0.409	**2.56E−15**	0.387	**1.18E−13**
SIGLEC9	0.559	**1.12E−29**	0.412	**1.64E−15**	0.562	**7.94E−30**
SIGLEC10	0.601	**3.81E−35**	0.512	**2.11E−24**	0.577	**1.01E−31**
SIGLEC11	0.251	**2.37E−06**	0.098	6.93E−02	0.363	**4.02E−12**
SIGLEC14	0.370	**1.27E−12**	0.220	**3.88E−05**	0.402	**1.11E−14**
SIGLEC15	0.119	**2.74E−02**	0.143	**7.86E−03**	0.001	9.93E−01
SIGLEC16	0.18	**8.17E−04**	0.122	**2.37E−02**	0.284	**9.10E−08**

Bold result means *P* value < 0.05

### Relationships between SIGELC family gene expression levels and clinicopathological characteristics of patients with HCC

**Fig. 8:**
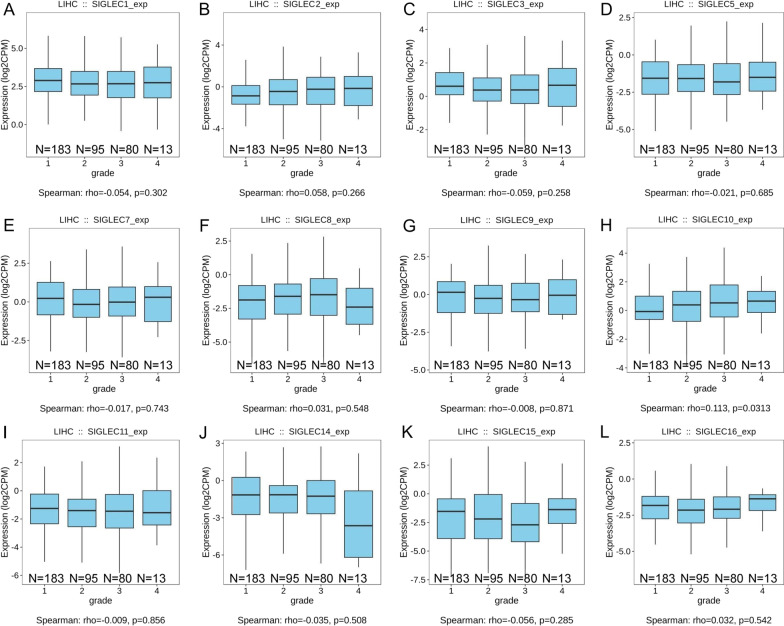
Correlations between SIGLEC mRNA expression levels and tumour grade in HCC. (TCGA database) **A–L**. *P < 0.05; **P < 0.01; ***P < 0.001

**Fig. 9:**
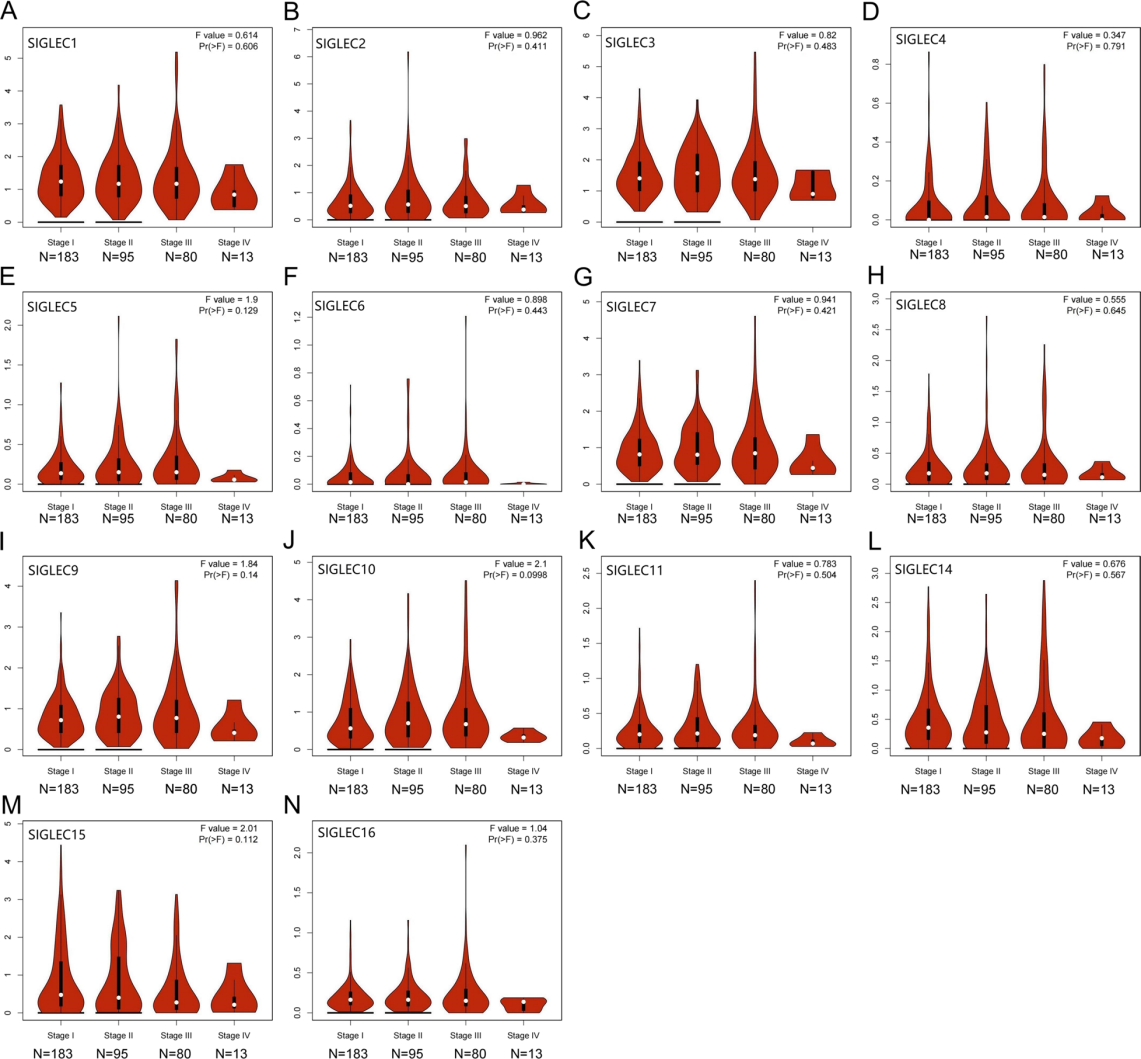
Correlations between SIGLEC mRNA expression levels and cancer stage in HCC. (TCGA database) **A–N**. *P < 0.05; **P < 0.01; ***P < 0.001

TCGA data were downloaded from GEPIA, UALCAN, and TISIDB. These data were used to analyse the correlations between SIGLEC mRNA expression levels and clinicopathological characteristics (including tumour grade and individual stage) in patients with HCC. SIGLECs family mRNA expression levels were correlated with cancer stage. Lower mRNA expression levels of SIGLECs corresponded with a worse tumour stage. Similarly, a worse tumour grade was related to lower mRNA expression levels of SIGLECs (Figs. 8, 9 and Additional file 1: Figs. S1–S2).

Overall, the mRNA expression levels of most SIGLEC family genes were correlated with the clinicopathological characteristics of patients with HCC.

### Mutations in SIGLEC family genes in HCC and correlations with prognosis

**Fig. 10:**
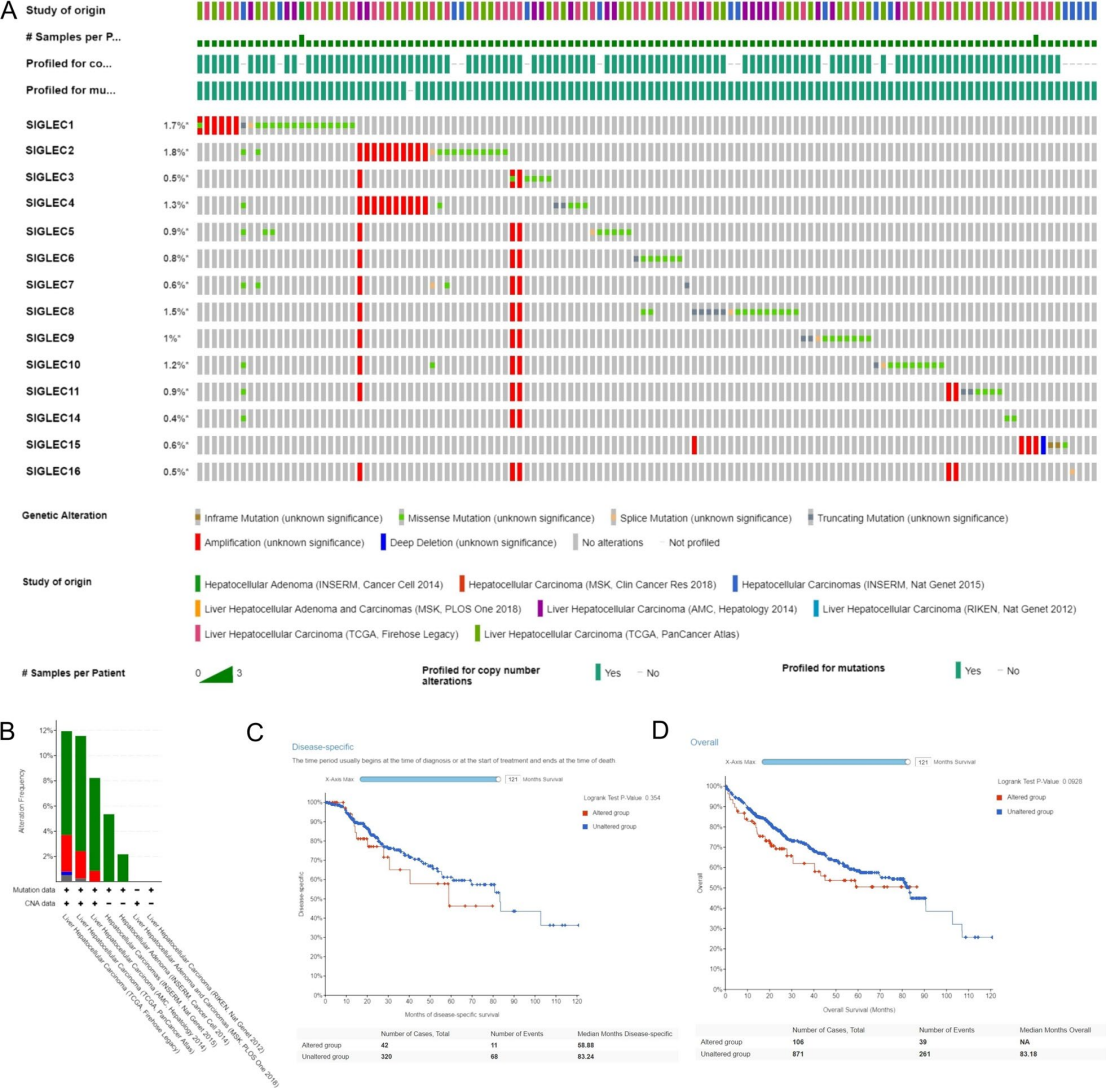
Type and frequency of SIGLEC gene mutations in HCC (cBioPortal database). **A** Overview of SIGLEC gene mutations.** B** SIGLEC gene alteration frequencies. **C** Significantly shortened disease-specific survival in the SIGLEC mutation group. **D** Significantly shortened overall survival in the SIGLEC mutation group

As shown in Fig. 10A, the type and frequency of SIGLEC family gene mutations in eight HCC datasets (containing 1507 samples) were explored using cBioPortal. The alteration frequencies in TCGA-Firehose Legacy, TCGA-PanCancer Atlas, INSERM-Nat Genet 2015, and AMC-Hepatology were 11.9%, 11.7%, 8%, 5.2%, and 2.1%, respectively (Fig. 10).

The SIGLEC family gene mutations were remarkably correlated with prognosis in HCC; the SIGLEC mutation group showed a significantly shorter OS and DSS than the wild-type group.

Overall, there was an important connection between SIGLEC family gene mutations and poor prognosis in HCC. Patients with HCC with normal expression of SIGLEC family genes are expected to have a better prognosis.

### Value of SIGLEC mRNA expression levels in predicting the prognostic of advanced HCC patients treated with sorafenib

**Fig. 11:**
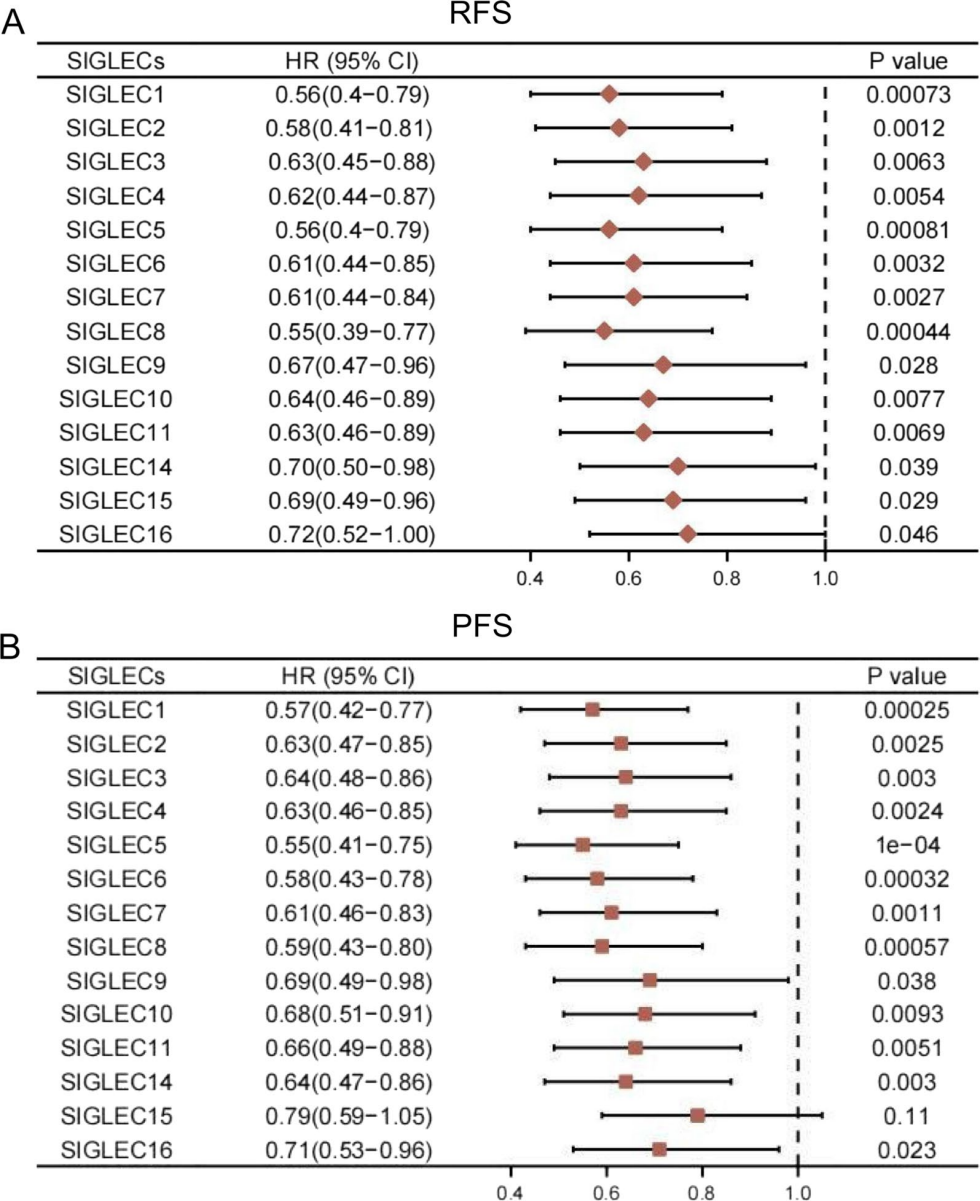
Forest plots of HRs for RFS and PFS with SIGLEC expression (Kaplan–Meier Plotter)

Kaplan–Meier Plotter was used to analyse the correlations between the mRNA expression levels of SIGLEC family genes and prognosis. Lower SIGLEC mRNA expression levels were significantly correlated with shorter RFS and PFS in advanced HCC patients (Fig. 11).

**Fig. 12:**
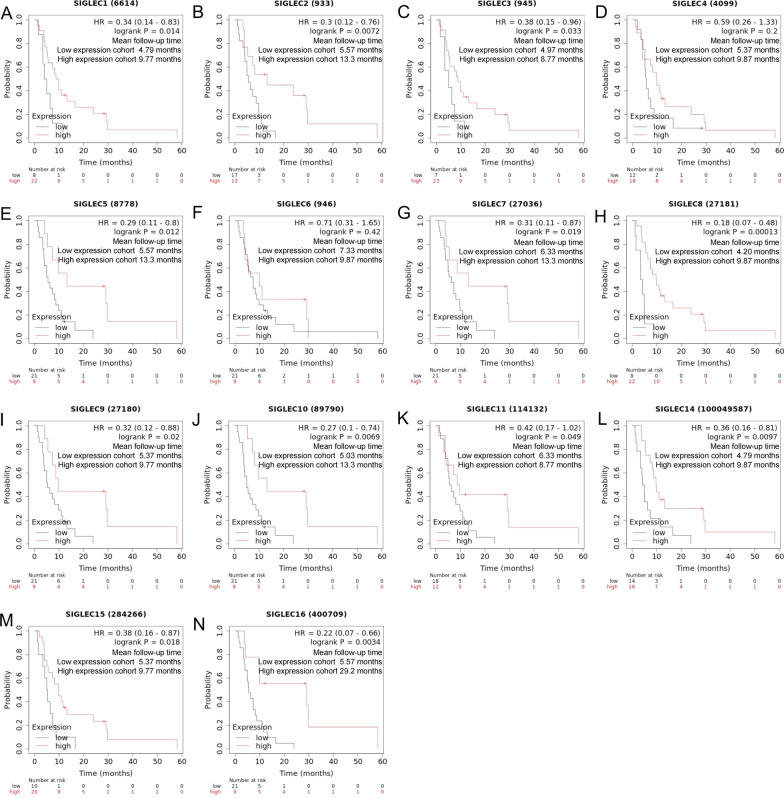
Effect of SIGLEC expression on survival and prognosis in patients with HCC treated with sorafenib (Kaplan–Meier Plotter)

In view of the lack of markers for the response to sorafenib, we analysed the expression levels of SIGLECs in patients with advanced HCC treated with sorafenib. Intriguingly, we found that the expression levels of SIGLECs were significantly correlated with the prognosis of patients with advanced HCC treated with sorafenib. High levels of SIGLEC expression were significantly associated with a better prognosis (Fig. 12 and Additional file 1: Figs. S3–S5).

The above results indicate that SIGLEC expression is correlated with prognosis in patients with advanced HCC treated with sorafenib. In the future, the SIGLEC family may become a predictive index for evaluating the efficacy of sorafenib, which is very important for the prognostic evaluation of patients with advanced HCC treated with sorafenib.

## Discussion

HCC has a poor prognosis and a high mortality worldwide, with an increasing number of cases every year [46, 47]. It is possible that the early stages of HCC could be cured. However, the diagnosis of early-stage HCC is difficult owing to the lack of obvious signs and diagnostic biomarkers [48]. Sorafenib prolongs the survival period of end-stage HCC by only several months [49]. Immunotherapy for HCC is still in its infancy compared to its application to other tumours [50]. Accordingly, HCC treatment is far from perfect. Therefore, it is imperative to explore therapeutic targets.

The functions of SIGLECs in the regulation of immune cell infiltration and cancer have been described by Macauley et al. [51]. In fact, SIGLECs are a family of sialic acid immunoglobulin receptors involved in immune discrimination. These receptors can regulate immune cells, such as T cells and B cells, in the adaptive and innate immune systems via their glycan ligands [51]. More recently, a report proposed that SIGLEC family genes in tumour cells are related to carcinogenesis [20]. Immune cell infiltration plays crucial functions in HCC progression [52]. Another study showed that CD24-SIGLEC10 interaction may be involve in immune responses[16] and reduced SIGLEC7 expression could cause NK cell dysfunction in HCC patients[17]. We observed strong correlations between the expression levels of SIGLECs and tumour-infiltrating lymphocytes. Taken together, these results indicate that SIGLECs are potential targets for cancer immunotherapy. The mechanisms by which SIGLECs interact with T cells and B cells in immune surveillance and immunoediting have been summarized previously [53]. Additionally, SIGLEC15 has been identified as an emerging target for immunotherapy [54]. Barkal et al. reported that CD24 is highly expressed and is related to the inhibitory receptor SIGLEC10 expressed by tumour‐associated macrophages to promote immune evasion in breast cancer and ovarian cancer [55]. Additionally, SIGLEC9 is involved in the innate immune response to cancer [56]. There are many studies of the relationships between SIGLEC family members and prognosis in patients with malignant tumours. Kensuke et al. observed a relationship between SIGLEC7 and prognosis in CRC; they found that the expression of SIGLEC7 in macrophages may become a novel prognostic biomarker for the efficacy of immunotherapy against metastatic CRC [18]. An in-depth understanding of the roles of SIGLECs in immune cell functions is expected to accelerate the development of cancer inhibitors [51].

Our results showed that the mRNA expression levels of the majority of SIGLECs genes were remarkably reduced in HCC tissues based on TCGA and GEO datasets. We obtained similar results at the protein level based on data from the HPA. Furthermore, we identified additional proteins, e.g., TNR, PTPN11, PLP1, CD19, PSG1, PSG2, and PSG7, related to SIGLEC by network analysis using GeneMANIA. The predicted biological functions of SIGLECs mainly included biological adhesion, neutrophil activation, and primary cell adhesion. These results were consistent with the molecular pathway correlated with HCC development. In an analysis of the associations of SIGLEC expression with clinicopathological features of patients with HCC, lower gene expression levels were correlated with a worse tumour grade.

High SIGELC family gene expression was remarkably correlated with a better prognosis in patients with HCC undergoing treatment with sorafenib. This improved prognosis may be explained by the relationship between high SIGLEC expression and the activation of the immune system. Sorafenib has several targets, including VEGFR, PDGFR, and RAF [57]. Additionally, SIGLEC5 may be related to VEGFR [58]. However, the interaction between these factors in improving prognosis needs to be further explored. In addition, the aetiology of liver disease, presence of cirrhosis, alpha-fetoprotein levels, tumour burden, and other factors affect the response to sorafenib. For example, high AFP and NLR have been identified as prognostic factors for poor OS in patients treated with sorafenib [59]. Therefore, future research aimed at the development of effective indicators should incorporate these variables.

Combination strategies involving sorafenib and immunotherapy are promising methods and are gaining attention in HCC research. It is now clear that SIGLEC family members function in the regulation of natural immunity. Our results reveal the molecular mechanism and prognostic significance of SIGLECs in HCC, providing a basis for the development of therapeutic and diagnostic strategies. Surprisingly, we found that SIGLECs are effective prognostic markers for the response to sorafenib in patients with advanced HCC; therefore, our findings have important clinical significance. Therefore, we explored the dual effects of SIGLEC family members to overcome the current limitations of HCC therapy. However, the predictive value of SIGLECs for the response to new first-line therapies (atezolizumab + bevacizumab) requires further research and exploration.

## Conclusion

In general, our results contribute substantially to research on the roles of SIGLECs as prognostic markers and anti-HCC therapeutic targets. Our study also provides an important theoretical basis for the clinical value of SIGLECs as a prognostic marker for HCC patients treated with sorafenib.

## Supplementary Information

**Additional file 1.** Further analysis of SIGLEC family expression and its relationship with patient survival.

## Data Availability

The datasets analysed in this research are available from the corresponding author. For the usage of R language, please contact the corresponding author. The accessible links of all the publicly available databases used in the study are as follows. The TCGA database is available at https://portal.gdc.cancer.gov/. The GEPIA database is available at http://gepia.cancer-pku.cn/. The Kaplan–Meier plotter is available at http://kmplot.com/analysis/. cBioPortal is available at https://www.cbioportal.org/. TIMER is available at https://cistrome.shinyapps.io/timer/. Linkedomics is available at http://linkedomics.org/. The HCCDB is available at http://lifeome.net/database/hccdb/.

## Abbreviations

AFP: Alpha fetoprotein
BP: Biological process
CAMs: Cell adhesion molecules
CC: Cellular component
CRC: Colorectal cancer
GEPIA: Gene Expression Profiling Interactive Analysis
GEO: Gene Expression Omnibus
GO: Gene Ontology
GTEx: Genotype-Tissue Expression
HBV: Hepatitis B virus
HPA: Human Protein Atlas
HCC: Hepatocellular carcinoma
HCCDB: Integrative Molecular Database of Hepatocellular Carcinoma
ICGC: International Cancer Genome Consortium
KEGG: Kyoto Encyclopedia of Genes and Genomes
MF: Molecular function
OS: Overall survival
ORA: Over-Representation analysis
PFS: Progression-free survival
RFS: Recurrence free survival
SIGLECs: Sialic-acid-binding Ig-like lectins
TIMER: Tumour Immune Estimation Resource
TCGA: The Cancer Genome Atlas
