# Supplementary material for: A comprehensive computational analysis to explore the importance of SIGLECs in HCC biology
Source: BMC Gastroenterol. 2023 Feb 18;23:42. doi: 10.1186/s12876-023-02672-z (PMC9938566; doi:10.1186/s12876-023-02672-z)
Supplement: Supplementary file 1 — Additional file 1. Further analysis of SIGLEC family expression and its relationship with patient survival. [file 12876_2023_2672_MOESM1_ESM.doc]

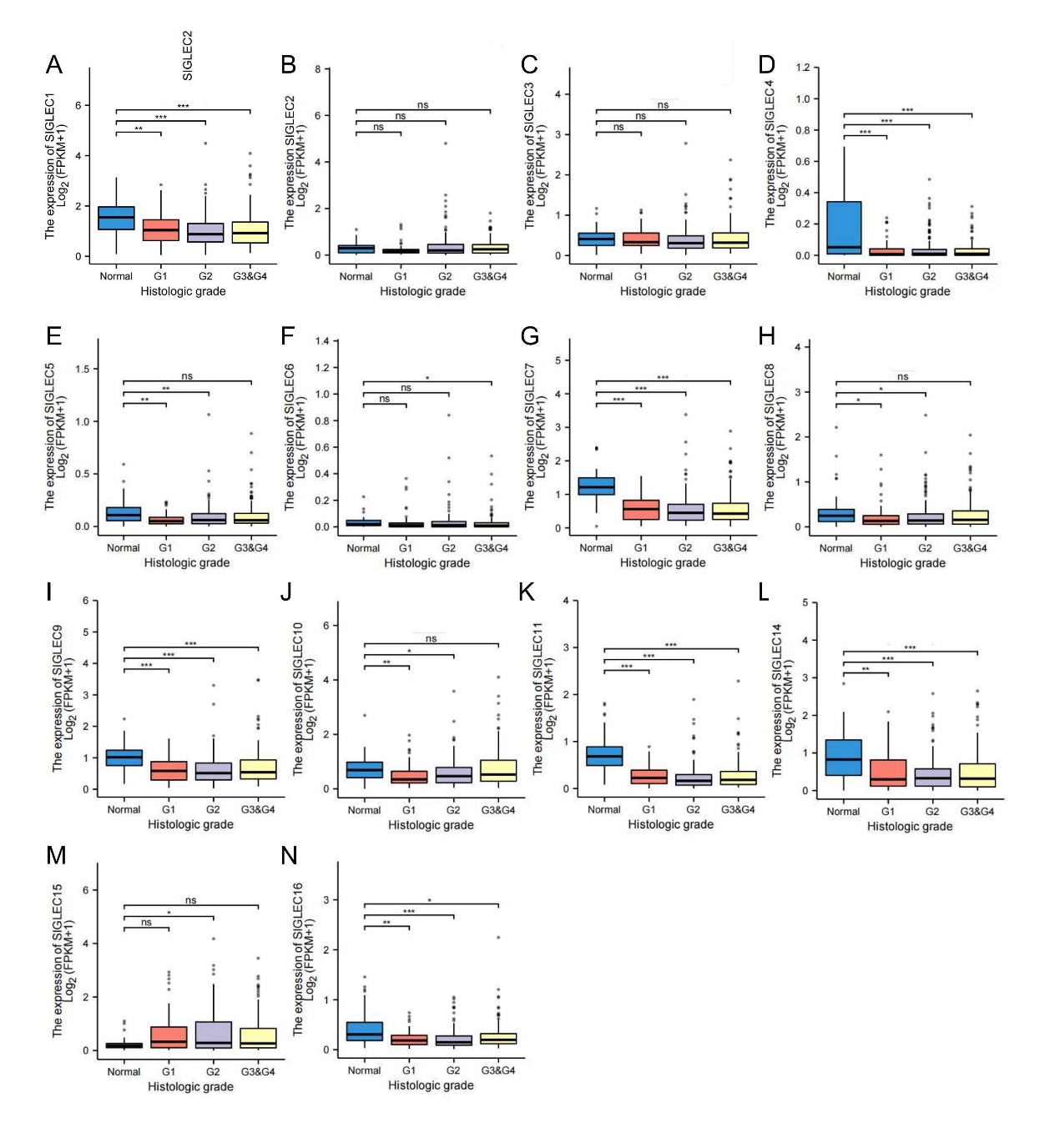


**Fig. S1 Correlations between SIGLECs mRNA expression levels and tumor grade in HCC. *P < 0.05. **P < 0.01. ***P < 0.001.**


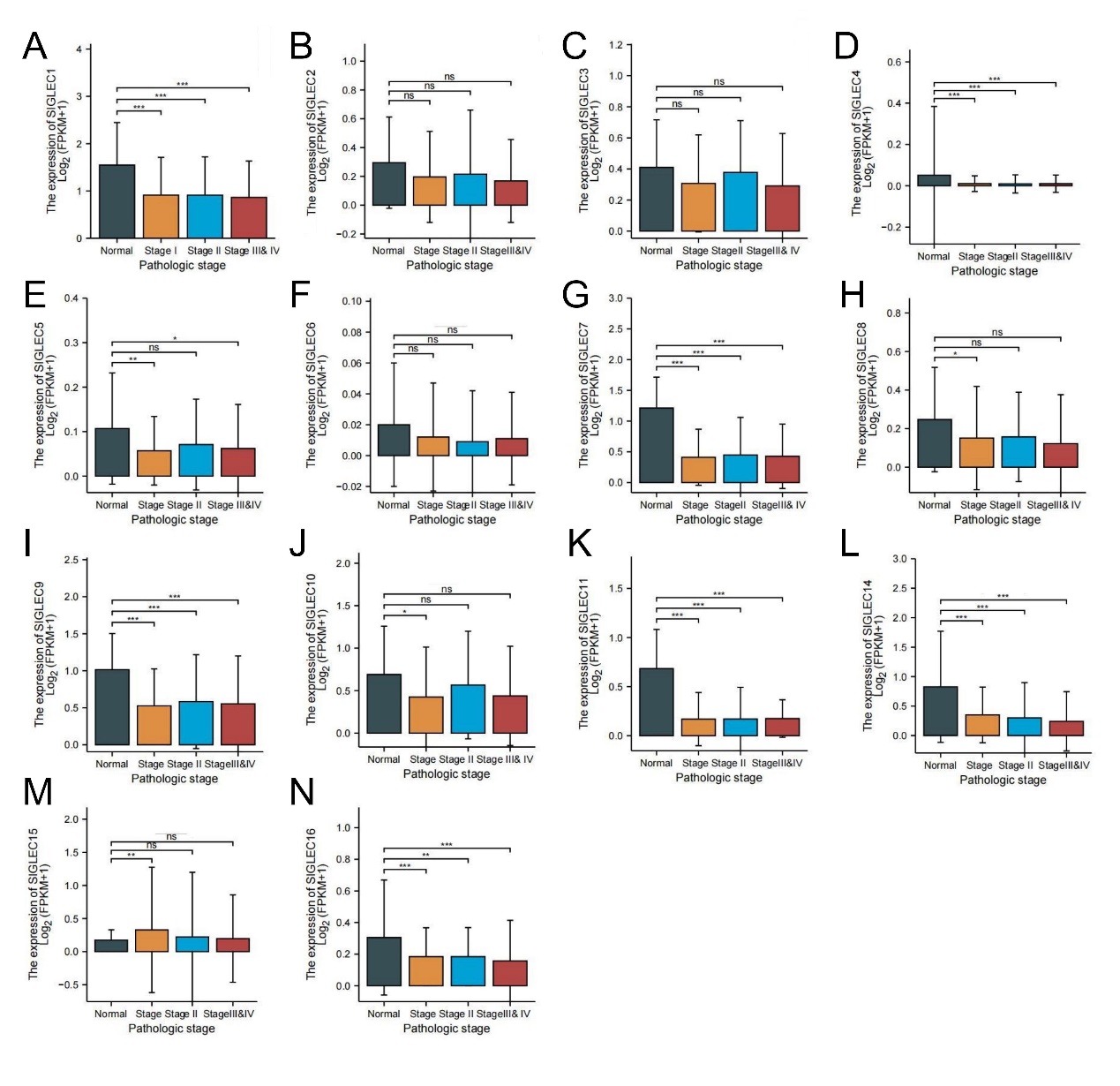


**Fig. S2 Correlations between SIGLECs mRNA expression levels and cancer stage in HCC. *P < 0.05. **P < 0.01. ***P < 0.001.**


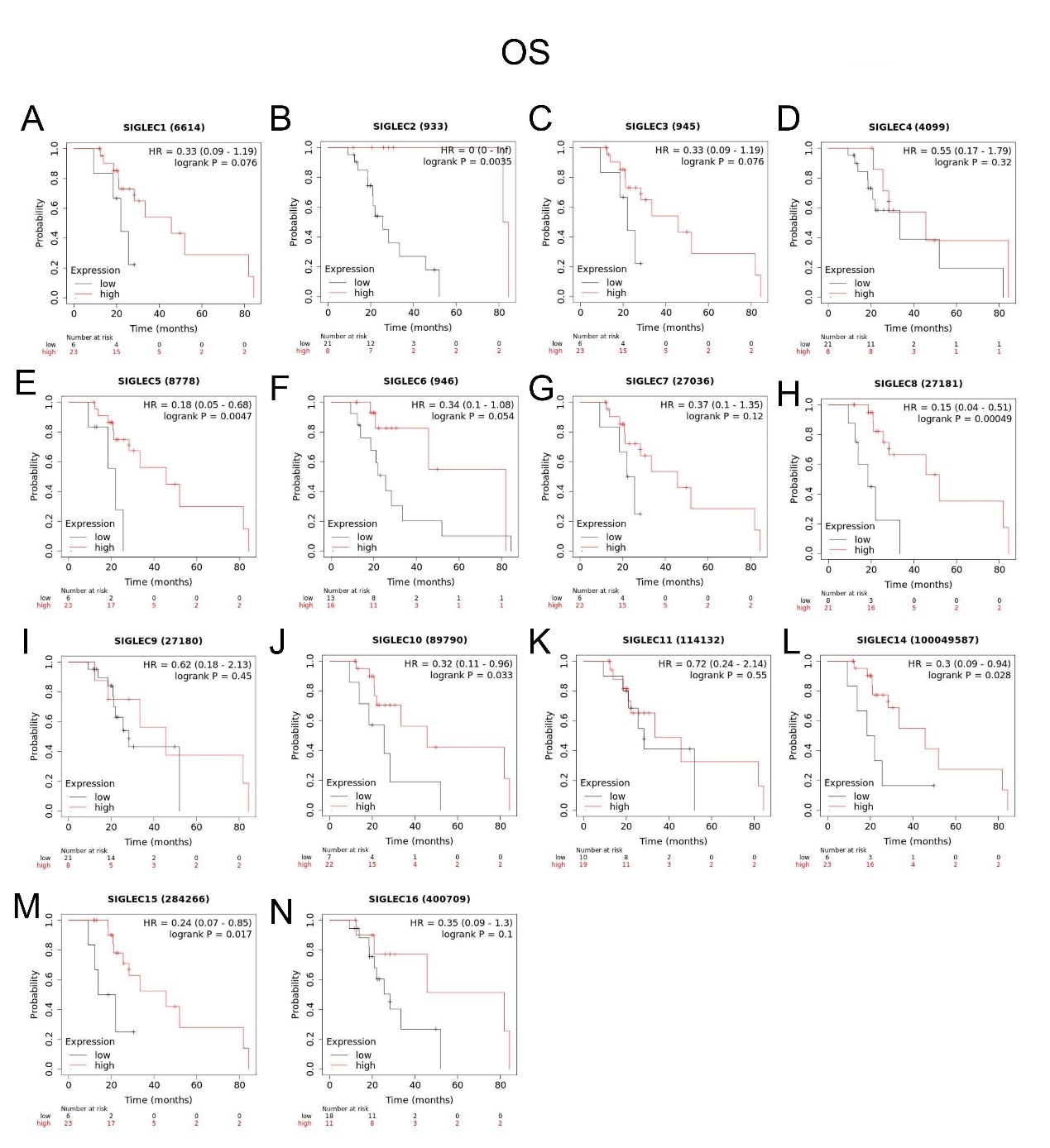


**Fig. S3 Effect of SIGLECs expression on OS survival in patients with HCC treated with sorafenib. (Kaplan-Meier Plotter).**


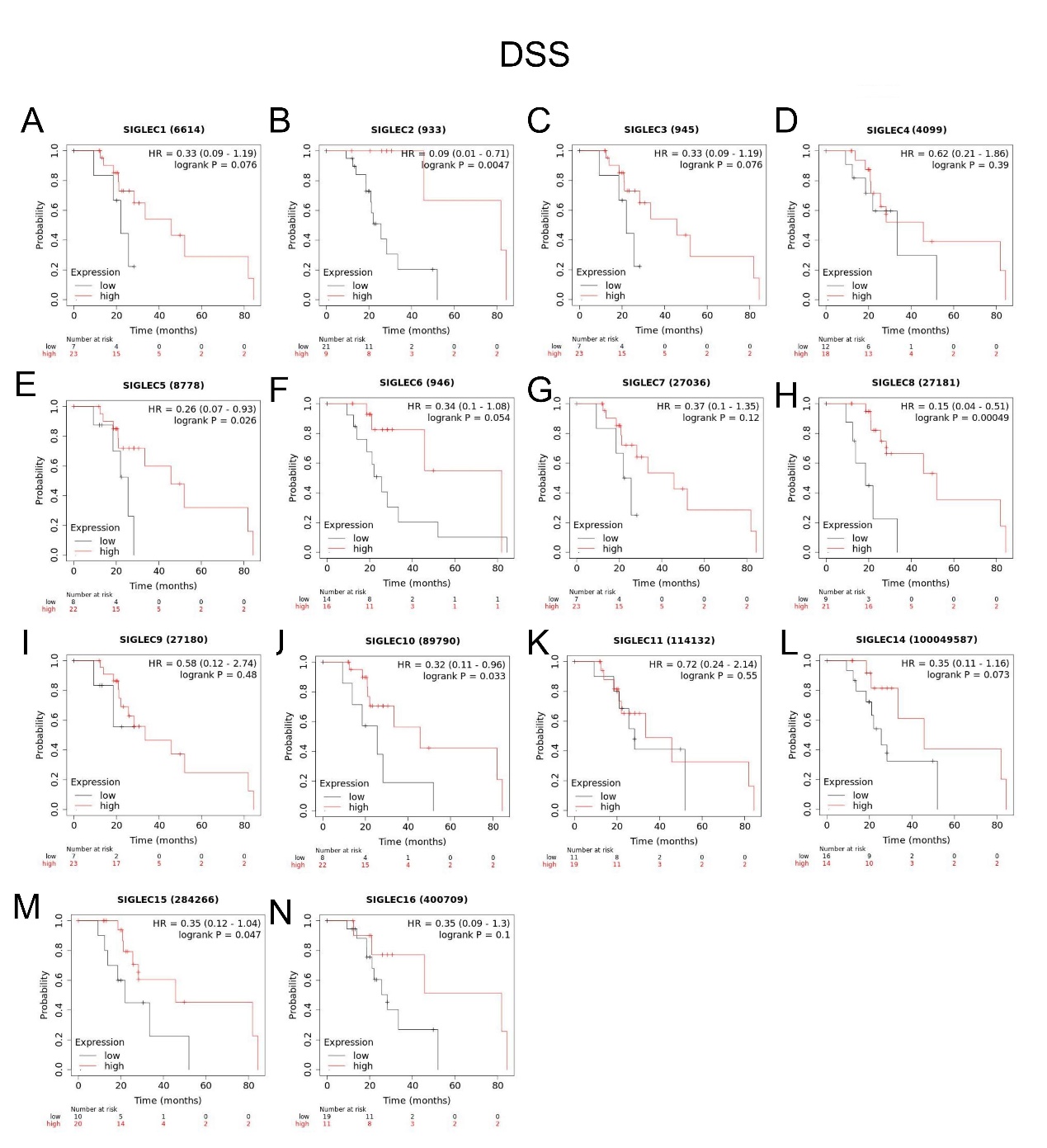


**Fig. S4 Effect of SIGLECs expression on DSS survival in patients with HCC treated with sorafenib. (Kaplan-Meier Plotter).**


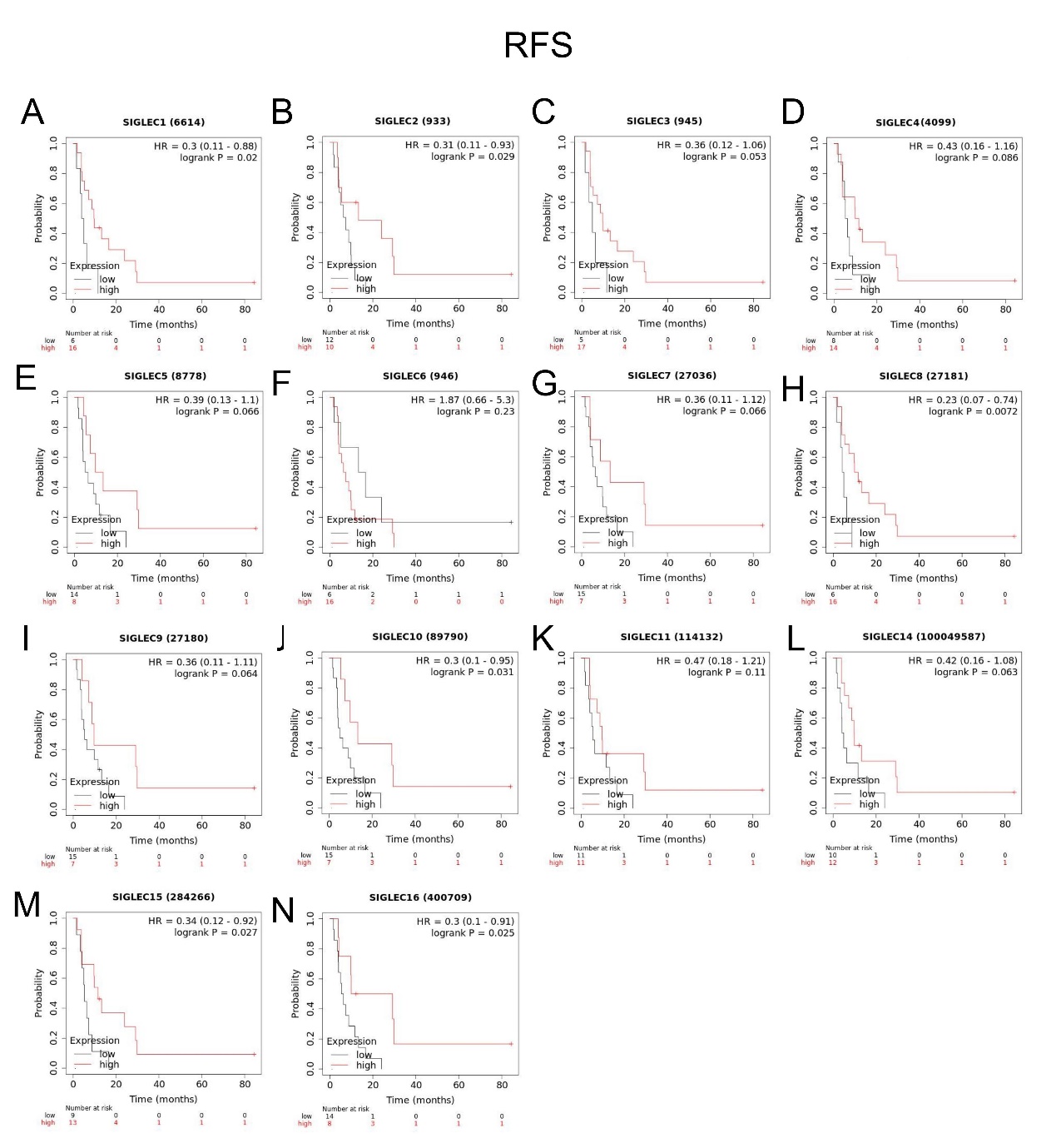


**Fig. S5 Effect of SIGLECs expression on RFS survival in patients with HCC treated with sorafenib. (Kaplan-Meier Plotter).**


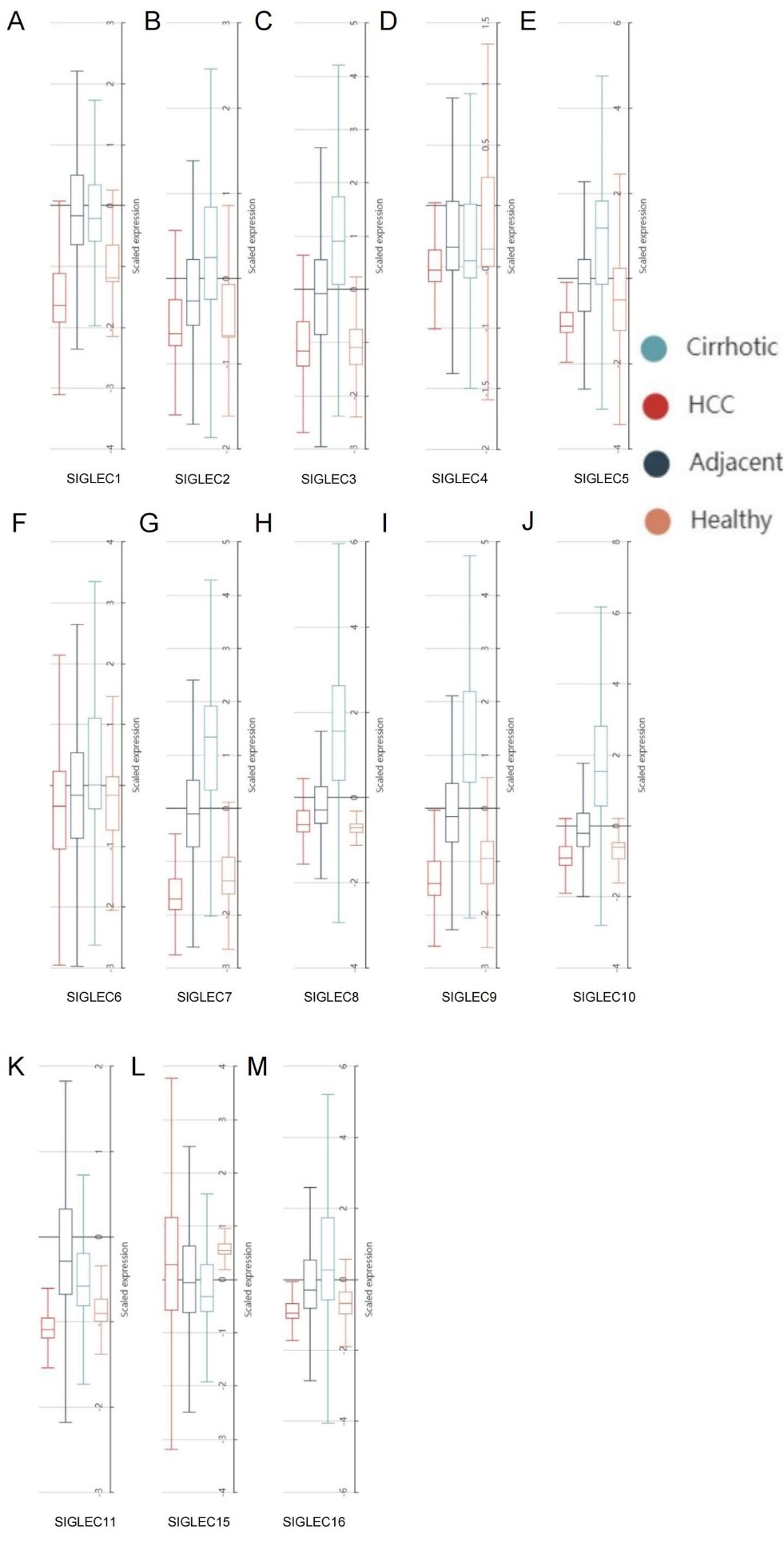


**Fig. S6 SIGLECs expression in cirrhotic controls, normal healthy controls, adjacent normal controls and HCC (GSE25097).**
